# Two‐point‐NGS analysis of cancer genes in cell‐free DNA of metastatic cancer patients

**DOI:** 10.1002/cam4.2782

**Published:** 2020-01-28

**Authors:** Maria Palmieri, Margherita Baldassarri, Francesca Fava, Alessandra Fabbiani, Elisa Gelli, Rossella Tita, Pamela Torre, Roberto Petrioli, Theodora Hadijstilianou, Daniela Galimberti, Elisa Cinotti, Carmelo Bengala, Marco Mandalà, Pietro Piu, Salvatora Tindara Miano, Ignazio Martellucci, Agnese Vannini, Anna Maria Pinto, Maria Antonietta Mencarelli, Stefania Marsili, Alessandra Renieri, Elisa Frullanti

**Affiliations:** ^1^ Medical Genetics University of Siena Siena Italy; ^2^ Genetica Medica Azienda Ospedaliera Universitaria Senese Siena Italy; ^3^ Oncology Azienda Ospedaliera Universitaria Senese Siena Italy; ^4^ Department of Ophthalmology Referral Center for Retinoblastoma Azienda Ospedaliera Universitaria Senese Siena Italy; ^5^ Department of Medical, Surgical and Neurosciences Dermatology Unit University of Siena Siena Italy; ^6^ Medical Oncology Ospedale Misericordia Azienda Toscana Sud‐Est Grosseto Italy; ^7^ Department of Otology and Skull Base Surgery University of Siena Siena Italy; ^8^ VisMederi s.r.l Strada del Petriccio e Belriguardo Siena Italy

**Keywords:** cell‐free DNA, liquid biopsy, next‐generation sequencing, solid tumors, targeted‐therapy

## Abstract

**Background:**

Although the efficacy of molecularly target agents in vitro, their use in routine setting is limited mainly to the use of anti‐HER2 and antiEGFR agents in vivo. Moreover, core biopsy of a single cancer site may not be representative of the whole expanding clones and cancer molecular profile at relapse may differ with respect to the primary tumor.

**Methods:**

We assessed the status of a large panel of cancer driver genes by cell‐free DNA (cfDNA) analysis in a cohort of 68 patients with 13 different solid tumors at disease progression. Whenever possible, a second cfDNA analysis was performed after a mean of 2.5 months, in order to confirm the identified clone(s) and to check the correlation with clinical evolution.

**Results:**

The approach was able to identify clones plausibly involved in the disease progression mechanism in about 65% of cases. A mean of 1.4 mutated genes (range 1‐3) for each tumor was found. Point mutations in *TP53*, *PIK3CA*, and *KRAS* and copy number variations in *FGFR3* were the gene alterations more commonly observed, with a rate of 48%, 20%, 16%, and 20%, respectively. Two‐points‐Next‐Generation Sequencing (NGS) analysis demonstrated statistically significant correlation between allele frequency variation and clinical outcome (*P* = .026).

**Conclusions:**

Irrespective of the primary tumor mutational burden, few mutated genes are present at disease progression. Clinical outcome is consistent with variation of allele frequency of specific clones indicating that cfDNA two‐point‐NGS analysis of cancer driver genes could be an efficacy tool for precision oncology.

## Graphical abstract

Core biopsy of a single cancer site may not be representative of the whole expanding clones and cancer molecular profile at relapse may differ with respect to the primary tumor. In this study, we performed a first and whenever possible, a second NGS liquid biopsy analysis after a mean of 2.5 months in advanced cancer patients. The approach was able to identify clones plausibly involved in the disease progression mechanism in about 65% of cases. Clinical outcome is consistent with variation of allele frequency of specific clones indicating that cfDNA two point‐NGS analysis of cancer driver genes could be an efficacy tool for precision oncology.

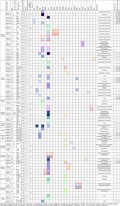

## 1 INTRODUCTION

Cancer cells continuously acquire new mutations due to genomic instability and/or selective pressure from the tissue microenvironmental and clinical treatment. During anti‐cancer drug treatment, subclones survive and multiply, contributing to further evolution of metastases into diverse tumor cell phenotypes. Several studies demonstrated that at disease progression, expanding clones are different with respect to those identified at the beginning in tumor biopsy and that expanding clones may be selected by progresses therapies^1^ and have differential sensitivities to therapy.^2^ This was extensively shown for both hematological^3^, ^4^, ^5^ and solid tumors.^1^

Large‐scale studies demonstrated a limited usefulness of molecular profiling obtained from Formalin‐Fixed and Paraffin‐Embedded tumor specimen of primary tumor, relapse, or metastasis.^6^ Tumor biopsies normally accomplish the sampling of only a part of the tumor and may only capture a fraction of its heterogeneity, consequently not being totally informative about the levels of genetic variability of a patient's cancer. Moreover, it is unlikely for a patient to undergo sequential biopsies of primary and metastatic lesions along tumor progression.^7^

During the last years, to answer the need of a more accessible approach for tumor genetic analysis, “liquid biopsy” is emerging as an innovative, minimally invasive and efficient alternative to investigate cancer cells being able to take multiple blood samples over time informing on what type of molecular changes are taking place in a tumor.^8^, ^9^, ^10^ Now the cell‐free DNA (cfDNA) analysis has the possibility to overcome the space‐time profile constraint of physical biopsies and opens a new scenario for personalized treatment. The usefulness of cfDNA sequencing for identifying markers of disease progression is well established.^11^, ^12^ The European Medicines Agency (EMA) in 2015, and the Food and Drug Administration (FDA) in 2016 approved the use of cfDNA extracted from plasma for detection of *EGFR* mutations in non‐small‐cell lung cancer (NSCLC) patients without tissue available or after resistance to a first or second generation TKIs.^13^ Notwithstanding the potential game‐changing role of cfDNA assessments, its clinical utility is still under investigation and translational trials focused on the impact of its integration in the therapeutic algorithm are pivotal and of great impact to further develop precision medicine approaches.

In the present study, we investigated whether combined cfDNA analysis may detect emerging clones and track the patterns of clonal dynamics in a case series of 68 metastatic cancer patients. We revealed that two‐point‐NGS (next‐generation sequencing) analysis in cfDNA is able to distinguish evanishing from expanding clones. Furthermore, we found that mutations in *TP53*, *PIK3CA*, *KRAS,* and *FGFR3* were the most commonly observed in solid tumor irrespective to the primary tumor type, opening the way to a history or‐free new era. This in turn could result in an innovative trial design. Finally, we showed that only by combining cfDNA analysis with genomic analysis, it is possible to distinguish the germline mutation/somatic mosaicism from the true expanding clones eventually responsible for disease progression.

## 2 MATERIALS AND METHODS

### 2.1 Patients

This is a 12‐months prospective study from March 2018 to March 2019, conducted at Medical Genetics Unit of the Azienda Ospedaliera Universitaria Senese (AOUS), Siena, Italy, for diagnostic purposes. Sixty‐eight patients with different solid tumors who experienced disease progression after standard therapy were enrolled in both pediatric and adult Oncology Clinics of AOUS and Azienda Toscana Sud‐Est, Italy. Patients were previously treated in advanced/metastatic setting and most of them were not eligible for a curative treatment. This study was consistent with Institutional guidelines and approved by the ethical committees of Azienda Ospedaliera Senese, Siena. Informed consent was obtained from the patient. Written informed consent for genetic analysis was obtained for all patients at the Medical Genetics Unit of the Azienda Ospedaliera Universitaria Senese, Siena, Italy.

### 2.2 Study subject

Inclusion criteria included patients with either locally advanced or metastatic solid tumor independently from the primary tumor site. Patients were excluded if they had early‐stage solid tumors. The main information collected for each patient includes, in addition to oncological data, genealogic tree and cancer family history on a genetic consultation setting.

### 2.3 cfDNA and genomic DNA sampling

A first peripheral blood sample for cfDNA analysis was either taken from medical oncology or during the genetic counseling visit at the stage of disease progression (R1). Plasma was used for cfDNA extraction while cell containing phase (buffy coat) was used for genomic DNA (gDNA) extraction using MagCore HF16 (Diatech Lab Line, Jesi, Ancona, Italy). A second sample (R2) for cfDNA analysis was taken at the follow‐up visit. For a part of patients, the second sampling was not possible either because they died or because they entered at the end of the study time period.

### 2.4 CfDNA extraction

Peripheral blood samples (10 mL) were collected from each patient and placed into PAXgene blood ccfDNA tubes (Qiagen, Hilden, Germany). The plasma was obtained from a double centrifuge at 1900 g for 15 and 10 minutes and cfDNA was extracted from 4 mL of plasma using MagMAX cell‐free Total Nucleic Acid Isolation Kit (ThermoFisher Scientific), according to manufacturer's instructions. cfDNA quality and quantity was verified, respectively, using the Agilent™ High Sensitivity DNA Kit (Agilent Technologies) on Agilent2100 Bioanalyzer (Agilent Technologies) and Qubit™ dsDNA HS Assay Kits on Qubit 2.0 fluorometer (Invitrogen).

### 2.5 NGS sequencing on cfDNA

CfDNA sequencing was performed using Oncomine™ Pan‐Cancer Cell‐Free Assay (ThermoFisher Scientific) on Life Technologies Ion Proton sequencer (Life Technologies). This technology is able to identify various types of alterations, including single‐nucleotide variants, insertions/deletions, gene fusions, and copy number variations (CNV) present in genes linked to cancer (clinical actionable mutations) with a reportable range up to 0.05%. The sequencing analysis was performed using Ion Reporter Server System (Thermo Fisher Scientific).

### 2.6 NGS sequencing on genomic DNA

*gDNA* library preparation was performed according to the protocol of Life Technologies for individual germline mutation. The NGS sequencing was performed on Life Technologies Ion S5 sequencer (Life Technologies) and postrun analysis was conducted using the “coverageAnalysis” and “variantCaller” plug‐in on Torrent Server Suite (Life Technologies). Tissue analysed was mainly blood. In cases of suspected mosaicism additional tissues such as urine and salivary fluid were used.

### 2.7 Statistical analyses

Statistical analysis was carried out with R statistical software, version 3.6.0.^12^ Overall survival (OS) was performed using the Kaplan‐Meier method and the Cox proportional hazards analysis using the “survival” package in R.^14^, ^15^ The proportional hazards assumption was satisfied through Schoenfeld residuals (*ρ* = 0.1016042, *χ* ^2^ = 0.1041084 and *P* = .7469541). Median cfDNA plasma level as variable was used as the middle value for survival analysis. An increase of 20% from R1 to R2 was used as the cut‐off point for survival analysis. OS was defined as the time between the date of enrollment and the date of death or the date of last follow‐up. A *P* value < .05 was used as threshold for statistical significance.

Differences in clonal evolution (increased/ decreased mutational load) between patients at relapse phase (R) and patients at regression (G) or stationary (S) phase were tested by the Fisher's exact test.

## 3 RESULTS

### 3.1 Patients’ characteristics

From March 2018 to March 2019, a total of 68 patients with either locally advanced or metastatic solid tumor were considered eligible and included in the study (Table 1). The mean age at the first circulating tumor DNA (ctDNA) analysis was 58 years (range 1‐82 years); 63.2% of patients were females. Out of 68 patients harboring advanced cancer, 19 had breast cancer, 15 non‐small‐cell lung cancer, 6 patients had glioblastoma; 5 ovarian cancer; 4 patients had pancreatic cancer; 3 patients uterine, retinoblastoma, oral or gastric cancer; 2 patients had cholangiocarcinoma, colorectal cancer or Sezary syndrome (cutaneous lymphoma) and 1 soft tissue sarcoma of right infratemporal fossa. Six patients had microsatellite stable tumors and all other patients were not tested for microsatellite instability on the tumor. Among patients with distant metastatic disease, the visceral metastasis was the most common metastatic site (32.4%) followed by coexistence of both bone and visceral (19.1%) (Table 1). The median follow‐up of OS for all patients was 3.2 months (range 1‐15). At the time of survival analysis, death by tumor progression occurred in 12/68 (17.6%) patients.

**Table 1:** Characteristics of patients

Subject characteristics	Total (N = 68)	%
Median age (years‐range)	58 (1‐82)	—
Median follow‐up (years‐range)	4.5 (0.5‐14.4)	
Gender		
Male	25	36.8
Female	43	63.2
Primary site		
Breast	19	28
Lung	15	22.1
Glioblastoma	6	8.8
Ovarian	5	7.4
Pancreatic	4	5.9
Uterine	3	4.4
Retinoblastoma	3	4.4
Oral	3	4.4
Gastric	3	4.4
Cholangiocarcinoma	2	2.9
Colorectal	2	2.9
Sezary	2	2.9
Sarcoma	1	1.5
Metastatic site		
Bone	4	5.9
Visceral	22	32.4
Both (Bone and Visceral)	13	19.1
Local invasion	29	42.6
Median follow‐up (years‐range)	4.5 (0.5‐14.4)	
No. alive patients	56	82.4
No. dead patients	12	17.6

### 3.2 cfDNA load

**Figure 1:**
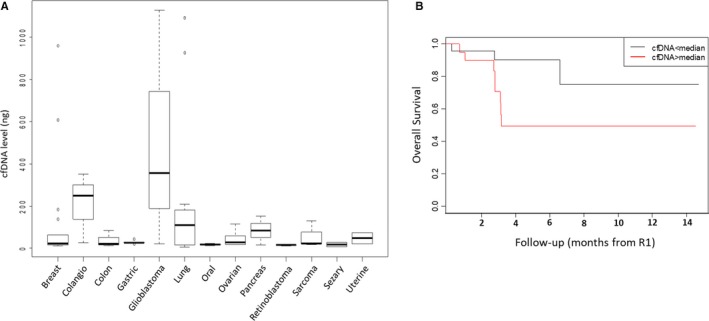
Plasma cfDNA levels according to primary tumor type and Kaplan‐Meier survival analysis. A, Box plot of cfDNA level (*y*‐axis) by primary tumor type (*x*‐axis). The line within each box represents the median fold‐change value. Upper and lower edges of each box, 75th and 25th percentile, respectively. Upper and lower bars, highest and lowest values determined, respectively. B, Kaplan‐Meier curve of OS according to cfDNA plasma level. CfDNA, circulating free DNA; OS, overall survival

Relative amount of cfDNA differs from patient to patient and from tumor to tumor, having lung cancer and glioblastoma the higher concentration (Figure 1A). In our case series of 68 metastatic cancer patients, the median cfDNA level at baseline (R1) was 27.2 ng (range 5.1‐1092) for 4 mL of plasma while the median cfDNA level at second liquid biopsy (R2) of 30.3 ng (range 5.91‐1128). The time span between R1 and R2 was an average of 2.4 month (range 1‐5 months, with only an out‐layer of 12 months).

In our case series, median OS was 3.2 months in the overall population. Use of Cox proportional hazard models for survival (adjusted for age) to evaluate the association between cfDNA levels and OS, showed that the risk of death was significantly higher for patients with high cfDNA amount [Hazard Ratio (HR): 4.81; 95% confidence interval (CI), 1.10‐21.09; *P* = .0372]. Kaplan‐Meier curves showed a statistically significant association between the cfDNA levels and OS (*P* = .043, Figure 1B).

### 3.3 Next‐generation sequencing analysis on cfDNA

**Figure 2:**
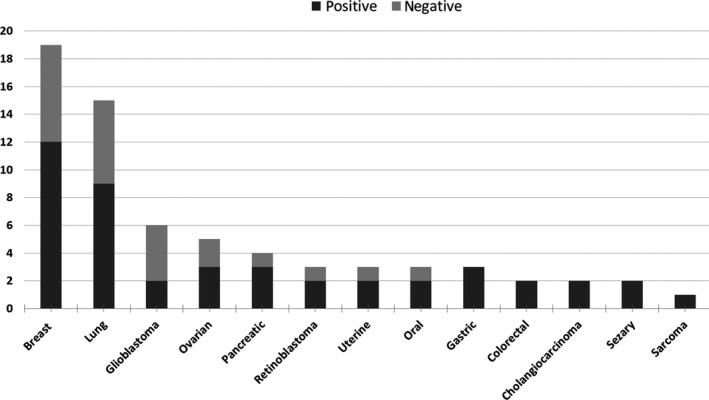
Distribution of 13 different tumor type in our cohort of 68 patients. Bar plot representing number of patients (y‐axis) grouped for primary tumor type (*x*‐axis). Number of positive cases (in dark gray) and negative cases (in light gray)

NGS analysis of 52 cancer genes of cfDNA samples of 68 patients allowed for picking up clones likely involved in the mechanism of disease progression in 65% of cases. The median follow‐up for positive cases was 2.9 months (range 0.3‐14.4). A mean of 1.4 mutated genes (range 1‐3) for each tumor was found. The percentage of positive cases was almost the same irrespective to primary tumor type (Figure 2).

**Figure 3:**
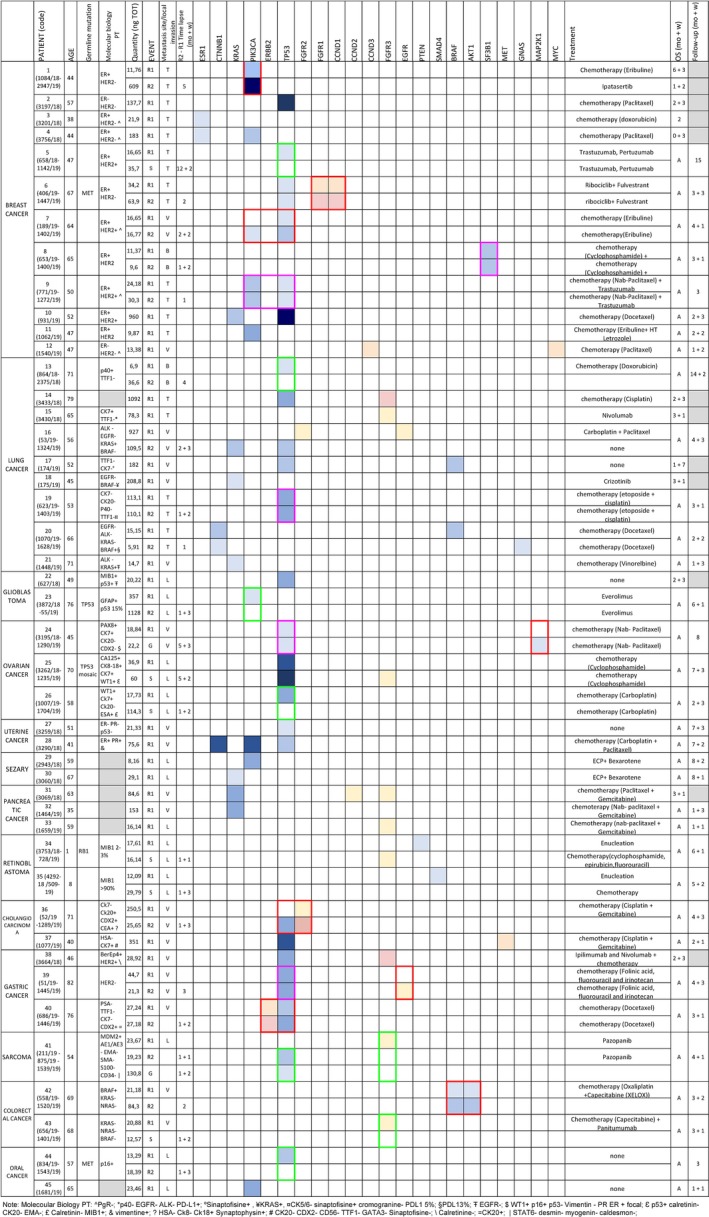
Clonal driver mutations identified in 68 patients with different solid tumors at disease progression. Driver mutations identified in 68 patients grouped for primary tumor type. Genes are represented on the top. Both CNVs (in pink) and SNVs (in blue) are indicated. Color scale indicates the variant allele frequency (VAF) of SNVs: from light [VAF ≤ 1%] to dark blue [VAF up to 50%]. The CNV ratio: from light [RATIO 1‐2] to dark pink [RATIO 10‐20]. *TP53*, *PIK3CA*, and *KRAS* and (CNV) in *FGFR3* were among the most commonly observed identified and persisting/growing clones, with a rate of 48%, 20%, 16% and 20%, respectively. Red boxes showed growing clones, fuchsia boxes showed stable clones and green boxes represented regression clones. In the event column R (relapse), G (regression), and S (stationary). In the metastasis site/local invasion column L (local), V (visceral), B (Bone) e T (both visceral e bone); Among 29 patients who received two‐point‐cfDNA analysis only 5 cases clearly showed growing clones (red outline). Most of them were mutation in 2 genes with parallel increasing of mutational load, suggesting double mutation of a single clone. For example, case 6 with *FGFR1/CCND1* clonal expansion in ER + HER2‐ breast cancer was treated by Ribociclib and Fulvestrant; case 40 with *ERBB2/TP53* clonal expansion in gastric cancer, in addition to chemotherapy, would benefit of Transtuzumab, which was not used due to advanced cardiopathy of the patient; case 36 with *FGFR2/TP53* clonal expansion in cholangiocarcinoma and case 42 with *BRAF/AKT1* clonal expansion in colorectal cancer are in the process to be treated by Erdafitinib and by Everolimus, respectively; and case 1 with *PIK3CA* clonal expansion in breast cancer was treated by Ipatasertib. Interestingly, retinoblastomas resistant to intra ocular Melphalan showed mutated clones in *PTEN* or *SMAD4* which disappeared after enucleation. One of the 2 tumors, the early onset with germline *RB1* mutation, started to grow a *FGFR3* clone after surgery indicating a incomplete disease remission

The comprehensive summary of mutations, including single‐nucleotide variants (SNVs) and copy number variants (CNVs), identified through cfDNA sequencing in each patient of our case series as well as treatments was represented in Figure 3. Clonal likely driver mutations in *TP53* were the most commonly observed along all patients regardless of the primary tumor type. A major Variant Allele Frequencies (VAFs) were observed for *TP53* and *PIK3CA* in breast and ovarian cancers (Figure 3).

**Figure 4:**
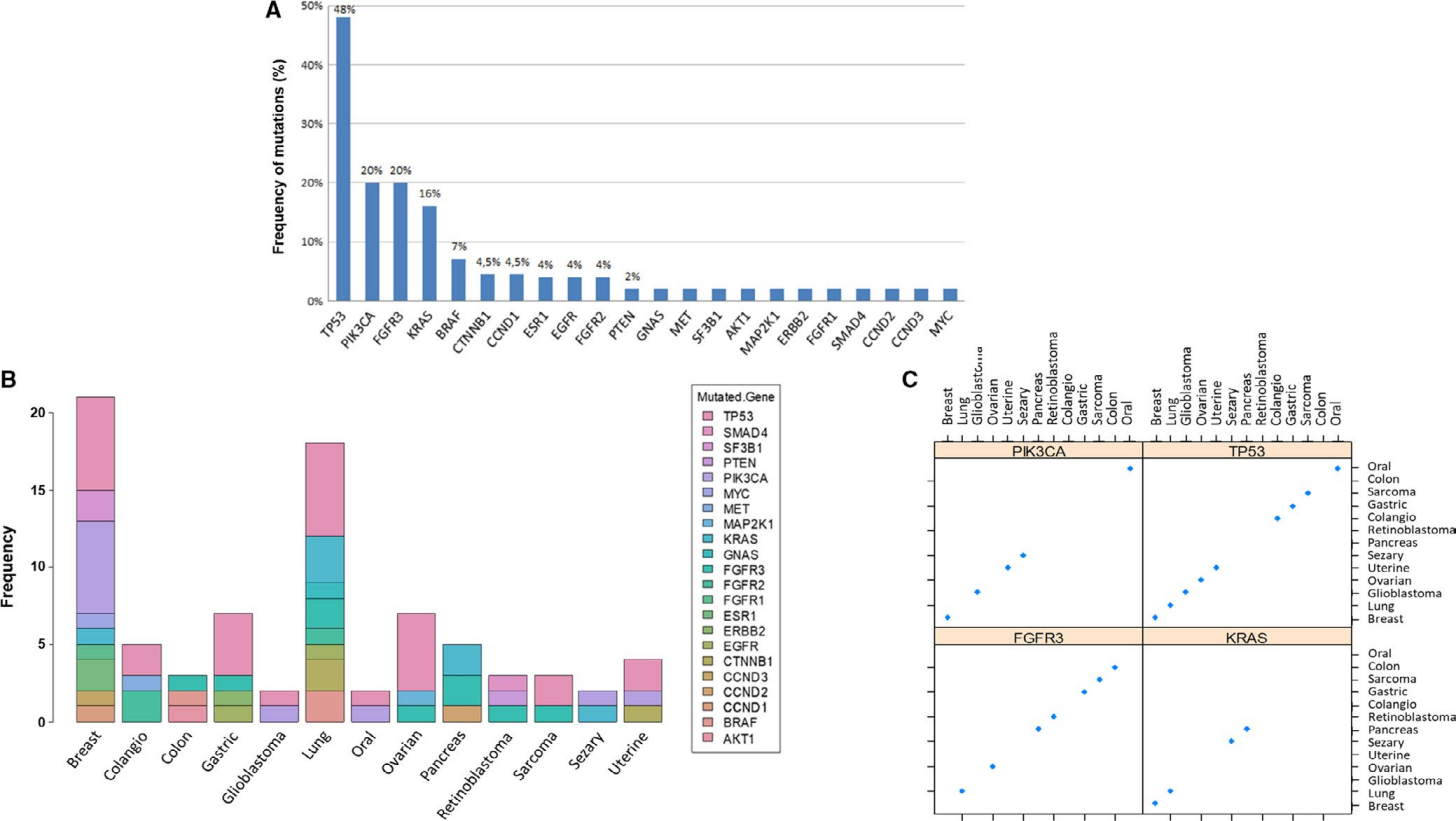
Distribution of the main alterations in mutated patients among the 68 included in the study. A, To establish the frequency of alterations within a gene, we considered the total number of alterations of that gene in the 45 positive patients, identified in the first analysis for these patients evaluated once (R1), in the second analysis in those evaluated twice (R2), because in the majority of cases, in particular 23/35, the second cfDNA analysis confirmed the clone identified in the first analysis. *TP53* is the most frequently observed clone (48%), followed by *PIK3CA* and *FGFR3* (20%). B, Bar plot showing the presence of mutated genes in frequency (*y*‐axis) accordingly to the primary tumor site (*x*‐axis). C, Scatter plot of mutations in the most frequently altered genes (*PIK3CA*, *TP53*, *FGFR3*, and *KRAS*) according to the tumor type

The distribution of all genomic alterations that were identified in the entire case series is shown in Figure 4. Point mutations in *TP53*, *PIK3CA*, and *KRAS* and CNVs in *FGFR3* were among the most commonly observed identified and persisting/growing clones, with a rate of 48%, 20%, 16% and 20%, respectively (Figure 4A). Overall mutations were detected in 22 different genes among 13 different solid tumors without specific prevalence (Figure 4B,C).

### 3.4 Germline next‐generation sequencing analysis

All those patients having a mutated clone close to 50% of mutational load were tested on DNA extracted from blood white cells. In 4 cases, one retinoblastoma, one glioblastoma, one breast cancer, and one oral cavity tumor, a germline mutation was identified; this was *RB1*, *TP53*, *MET*, and *MET*, respectively (Figure 3). In another case (ovary cancer), a *TP53* mutation in the mosaic state was identified (Figure 3).

### 3.5 Two‐point‐NGS analysis

**Figure 5:**
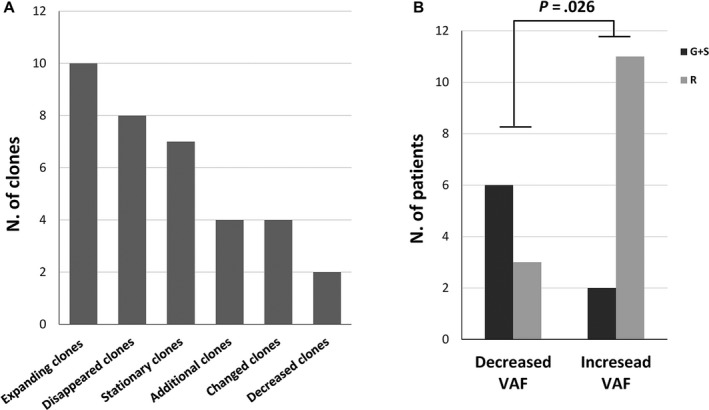
Clonal driver mutations evolution between first (R1) and second sample for cfDNA analysis (R2). A, Bar plot showing number of clones (*y*‐axis) according to clonal evolution of cancer driver genes between first (R1) and second sample for cfDNA analysis (R2) (*x*‐axis). If a sample showed different clones, each of them was counted. We see that the mutational load of ten clones was increased in the second sample with a mean time lapse R2‐R1 of 2.5 months while seven clones were stationary (R2‐R1: 2.2 months). Eight clones identified in the first analysis disappeared in the second one (R2‐R1 3.2) and just for two clones the mutational load was decreased with a mean time lapse R2‐R1 of 1.1 months. Four additional (R2‐R1 3.1 months) or changed (R2‐R1 1.9 months) emerging clones were identified in the second analysis. (B) The histograms show the distribution of the patients with decreased or increased variant allele frequency (VAF) between R1 and R2 according to clinical disease course (R, relapse; G, regression; S, stationary)

In the majority of cases, the second cfDNA analysis confirmed the clone identified in the first analysis (14/23, 61%) and among these, often (10/14, 71%) the variant allele frequency was increased (Figure 3). In other cases, the clones were stationary (Figures 3 and 5A). In few cases, additional emerging clones (4/35, 11.4%) were identified in the second analysis (R2‐R1 3.1 months), pinpointing to a minimal but still present clonal evolution (Figure 5A). In some cases, the clone identified in the first analysis (8/35, 23%) was not present anymore in the second (R2‐R1 2.5) and just in 2 cases the allele frequency was decreased with a mean time lapse R2‐R1 of 1.1 months (Figure 5A). Notably, the variant allele frequency increased in parallel with worsening of disease (Figure 5B). Overall, statistically significant association with clonal evolution was observed according to tumor burden. Patients with an increased or persistent mutational load at R2 reported significantly worse clinical outcomes compared with patients with decreased mutational load (*P* = .026, Figure 5B and Supplementary Table. 1).

Interestingly, among the disappearing clones, 62.5% were *TP53* mutated. In these cases, the variant allele frequency was sometimes not negligible, being around 5% to 13%.

## 4 DISCUSSION

More than 50% of solid cancers sooner or later escape control of standard treatments. Those tumors with high mutational burden are easily treated with immunotherapy, which is almost ineffective in another half of cases in which specific driver genes are supposed to lead therapy resistance.^16^ However, molecular‐based recommended therapies have almost unfulfilled expectation. In the majority of studies the molecular profiling is inferred from either primary tumor or metastases, none of them representing the evolving expanding clone at disease progression.^6^ ccfDNA is one tool capable to represent at once every metastasis and to follow them during the time showing clonal evolution of cancer driver genes which may play a major role in the therapy escaping mechanism.^11^

Previous studies indicate the amount of cfDNA as a prognostic factor.^17^ Results presented here confirmed the prognostic role of cfDNA plasma level demonstrating that the detection of more than 27.2 ng (per 4 mL of plasma) allowed to stratify the patient's’ overall survival. In our prospective case series, we observed a higher rate of death in the group of patients with cfDNA level > 27.2 at R1 compared with the group with cfDNA level < 27.2 (24% vs 11.8%). This difference became more evident when we observed only the subgroup of mutated patients (40% vs 12%).

Regarding the frequency of mutation in cfDNA, our data confirmed the literature evidences with SNVs in *TP53*, *PIK3CA* and *KRAS* and CNVs in *FGFR3* as the most commonly observed in breast and lung.^18^, ^19^ Our data demonstrate the absence of a correlation with mutation type and primary tumor organ since we identified mutation in 22 different genes among 13 different solid tumors without specific prevalence (Figure 4). The sniper clones leading to disease progression can be distinguished by cfDNA two‐point‐NGS analysis pinpointing the needs of grouping patients on truly growing clones instead of on primary tumor organ in clinical trials.

The results of our study showed that irrespective of the primary tumor mutational burden^20^ and subsequent complex clonal evolution, a simplified mutational load, in term of mutated clones, is present at disease progression (Figure 5). One or few “sniper” clones drive progression and the molecular profile of metastatic tumor has a weak correlation with the primary tumor. True snipers clones can be distinguished by cfDNA two‐point‐NGS analysis (Figure 5), highlighting the possibility to develop a specific therapy. For example gastric cancer with growing *TP53*/*ERBB2* mutated clone will benefit of Transtuzumab plus chemotherapy.^21^ Breast cancer with *CCND1*/*FGFR1* mutated clone will benefit of combined erdafitinib plus ribociclib and fulvestrant.^22^

In the majority of cases, the second cfDNA analysis confirmed the clone identified in the first analysis (Figure 3). However, not 100% of mutated clones were still present at the second liquid biopsy. For example *PIK3CA* clone in patient 1 is clearly progressive within 4 per months and correspond to clinical worsening. On the contrary, *TP53* in patient 5 disappeared and the patient is still alive after 12 months. In some cases (example case 6) one subclone expands faster than other, pinpointing as a possible main target. Therefore, the second cfDNA time point evaluation is mandatory to identified true targets for personalized medicine.

In this study, the time span between the first and the second liquid biopsy was an average of 2.5 months. Considering that 80% of clones has evolved at R2 and those not evolving had a time lapse about 1.5, this observation may suggest that a mean of 2.5 months could be the appropriate time lapse could be used in the clinical practice.

Driver mutations in *TP53* remain the main target of a not yet developed specific therapy in a wide range of progressing tumor such as breast, ovarian, uterine, lung, gastric cancers, oral, glioblastoma, and sarcoma (Figure 3). In many cases, the *TP53* mutations were accompanied by another mutation and 100% of *TP53* disappearing clones were alone. Overall, these data may suggest that *TP53* may act at “disease progression” as a main co‐driver gene, reducing apoptosis and cooperating with another cancer driver gene determining the growing of metastasis/local expansion. Indeed, clones with *TP53* mutation seems to be either stationary/disappearing when alone or growing with a second mutation.

Among the actionable mutations, *PIK3CA* were found not only in the very well known breast cancers but in a number of other cancers from like uterine carcinoma, Sezary syndrome, oral cancers, and glioblastoma, with the exception of lung. At the same time, increased CNV of *FGF* receptors were identified in patients with non‐small‐cell lung, pancreatic, and gastric cancer, and cholangiocarcinoma. These observations pinpoint the needs of trials grouping cancer patients on growing clones instead of primary tumor tissue/organ. Therefore, what we want to point out in this paper is that different histological tumors manifest the mutations onset in the same gene and sometimes the same variant. This new approach based on molecular features of cancer at disease progression, irrespective of the primary tumor origin, may be the keystone that directs towards a real personalized medicine.

Using a comparable number of tested genes the study of Rossi et al identified, in advanced breast cancer, the same average number of mutation per patient (3) but with a more wide variability (0‐27).^18^ One possible explanation of this discrepancy is the different cut off in considering variant as likely pathogenic. A second likely explanation could be assigned to lack of subtraction of germline mutation and somatic mosaicism, which are not tested in the Rossi's study. Now we know that germline mutations could be identified even in unsuspected sporadic cases as demonstrated here by the *TP53*‐mutated 76‐aged sporadic glioblastoma case, and *MET*‐mutated oral and breast cancer case. Somatic mosaicism is even more challenging to be identified and it can be testified only by multiple tissue analysis. One of the message that can be retrieved is that liquid biopsy could be always done in a setting in which comparison with genomic DNA is possible, which is usually genetic consultation.

Misinterpreting a germline mutation (which could appear in a percentage around 50 or more) or a somatic mosaicism (which could appear in any percentage below 50%) for a clonal mutation has serious practical consequences within personalized medicine. Germline mutation could be a useful target in early phase of the disease but somatic growing mutated clones are the main targets at disease progression. Targeting the germline mutation, although not dangerous itself, could not generate effect on outcome; hence somatic/germline misinterpretation could have fatal consequences for the patient leading in turn to underestimation of personalized medicine power.

To our knowledge, this is the first manuscript focusing on specific clonal evolution using at least two points of consecutive ctDNA analysis in a histotype unselected cohort of metastatic cancer patients. Figure 3 clearly shows that not 100% of mutated clones are still there at the second liquid biopsy. Failed personalized treatment toward those clones not persisting/not growing could be misinterpreted as failure of personalized medicine. Therefore, the second main take of message of this paper is to pay attention to start a personalized treatment only after a second cfDNA check confirming the presence/growing of targeted mutated clones.

## 5 CONCLUSIONS

In conclusion, our results indicate that cfDNA two‐point‐NGS analysis of cancer driver genes could be an efficacy tool for precision oncology. Indeed, the identification of key mutations that are responsible for tumor growth allows optimizing the therapeutic choice by addressing targeted therapy against specific driver mutation(s) of growing clones. This strategy may be the only one needed to win the war on individual patient.

## CONFLICT OF INTERESTS

The authors declare that they have no competing interests.

## AUTHORS' CONTRIBUTIONS

MP performed the experiments, analyzed the data, and wrote the paper, FF and AF took care of the clinical part of the study and wrote the paper, RT and EG performed experiments on blood, tumoral tissue, and metastasis. AR designed the research strategy, analysed the data, and wrote the paper, EF analysed the data and wrote the paper, MB, AMP, and MAM performed genetic counselling and provided patient samples. TH, DG, EC, and MM took care of the patients at first need. SM, CB, STM, IM, PT, RP, and AV were the oncologists.

## Data Availability

The data that support the findings of this study are available from the corresponding author upon reasonable request.
