# Supplementary material for: Two‐point‐NGS analysis of cancer genes in cell‐free DNA of metastatic cancer patients
Source: Cancer Med. 2020 Jan 28;9(6):2052–61. doi: 10.1002/cam4.2782 (PMC7064095; doi:10.1002/cam4.2782)
Supplement: Supplementary file 1 [file CAM4-9-2052-s001.docx]

| **Supplementary Table 1.** Patient details about clonal evolution (molecular) between R1 and R2 according to clinical disease course. | | |
| --- | --- | --- |
| **Patients** | **Molecolar** | **Clinical** |
| 5 | Absent | S |
| 13 | Absent | R |
| 23 | Absent | R |
| 26 | Absent | S |
| 43 | Absent | S |
| 44 | Absent | R |
| 41 | Absent | G |
| 8 | Stationary | R |
| 9 | Stationary | R |
| 19 | Stationary | R |
| 7 | Increased | R |
| 24 | Increased (+one stationary) | G |
| 39 | Increased | R |
| 1 | Increased | R |
| 6 | Increased | R |
| 36 | Increased | R |
| 40 | Increased | R |
| 42 | Increased | R |
| 25 | Increased | S |
| 35 | Stationary | G |
| *Note: patients 16, 20, and 25 were discarded because they were too complex in the molecular dynamics interpretation. Two patients (34, 35) with retinoblastoma were discarded because they were enucleated after the R1 event. R, relapse; G, regression; S, stazionary.* | | |
